# Assessment of Medical Students' Ability to Integrate Point-of-Care Cardiac Ultrasound Into a Case-Based Simulation After a Short Intervention

**DOI:** 10.7759/cureus.27513

**Published:** 2022-07-31

**Authors:** Frances M Russell, Audrey Herbert, Dina Peterson, Paul M Wallach, Robinson M Ferre

**Affiliations:** 1 Emergency Medicine, Indiana University School of Medicine, Indianapolis, USA; 2 Diagnostic Medical Sonography, Indiana University School of Medicine, Indianapolis, USA; 3 Internal Medicine, Indiana University School of Medicine, Indianapolis, USA

**Keywords:** clinical integration, cardiac ultrasound, simulation, undergraduate medical education, point of care ultrasound

## Abstract

Introduction: While a large amount of point-of-care ultrasound (POCUS) undergraduate medical education research exists, very little assesses the effectiveness of teaching on the student’s ability to utilize POCUS within a clinical context. We set out to assess the ability of pre-clinical (second year) medical students to perform and interpret a parasternal long axis (PSLA) cardiac ultrasound view, and to diagnose a pericardial effusion on POCUS in a simulated patient with hypotension.

Methods: This was a prospective study assessing second-year medical students before and after focused cardiac POCUS instruction. Pre-instruction, students completed a pre-assessment and test. They then watched a short video on cardiac ultrasound technique, anatomy, and pathology. Students then participated in 10 minutes of one-on-one hands-on instruction using a simulated patient. Immediately after didactics and hands-on instruction, students in groups of two to four completed a case simulation where they performed a PSLA view, identified pathology, and made a diagnosis. Differences between pre- and post-workshop responses were analyzed using the Chi-square test.

Results: We analyzed data on 132 pre-clinical second-year medical students; 126 (95%) had limited to no POCUS experience prior to the workshop. Comparing pre- to post-workshop responses, we found significant improvement in students’ ability to identify a pericardial effusion (46% to 69%) (p=0.002) on a PSLA cardiac view. Of the 57 student groups (132 students), 41 (72%) groups were able to adequately obtain a PSLA view on a mannequin using an ultrasound simulator without needing guidance with probe placement or maneuvering. Thirty-five (61%) student groups were able to identify a pericardial effusion and diagnose cardiac tamponade in a simulated patient with hypotension.

Conclusion: After short, structured training, pre-clinical medical students, novice to cardiac POCUS, showed improved knowledge with identifying a pericardial effusion on an ultrasound image. The majority of students were able to obtain a PSLA view and diagnose cardiac tamponade in a hypotensive patient during a during a case-based simulation.

## Introduction

Point-of-care ultrasound (POCUS) is an important tool that clinicians use at the bedside to quickly make a diagnosis and guide management decisions [1]. Most medical schools in the United States have implemented a POCUS curriculum to augment traditional learning [2-4]. The literature surrounding undergraduate medical education (UME) curriculum implementation has primarily focused on student attitudes towards POCUS, knowledge of POCUS, and skill with acquiring images [5-9]. Few studies have evaluated outcomes outside of this [10-14], including assessing the effectiveness of teaching on the student’s ability to utilize POCUS within a clinical context. 

We set out to assess if pre-clinical (second year) medical students, novice to POCUS and with limited clinical knowledge, could perform a parasternal long axis (PSLA) cardiac ultrasound view, interpret a pericardial effusion on POCUS, and diagnose cardiac tamponade in a simulated patient with hypotension. Secondarily, we assessed changes in comfort with and knowledge of performing cardiac POCUS before and after structured training.

## Materials and methods

Study design and participants

This was a prospective study, conducted on 132 second-year medical students during their physical examination course, assessing their ability to learn and perform a limited POCUS cardiac examination and diagnose a pericardial effusion on a simulated patient immediately after participating in a short, focused didactic, and hands-on POCUS instruction session. Students completed a pre- and post-POCUS workshop assessment, and a post-workshop simulated case. Data were collected over two days in August 2020. This study was approved by Indiana University, Indianapolis, United States (approval no 11394), with a waiver of informed consent.

Study protocol

The pre-assessment consisted of four questions and assessed prior experience with general POCUS and comfort with performing cardiac POCUS. These questions were adapted from earlier studies assessing novice learners [15]. There were two multiple choice questions evaluating image acquisition and PSLA image interpretation. The post-assessment consisted of three questions and re-assessed comfort, image acquisition, and interpretation.

Pre-lab and as part of the curriculum taught during their cardiovascular and hematology block course within the second year of medical school, students were required to asynchronously watch a 25-minute video that reviewed sonographic cardiac anatomy, how to acquire images for a focused cardiac ultrasound examination, pathology including recognizing signs of tamponade, acute left heart failure, and pulmonary embolism and indications for when to perform an exam clinically. The video also reviewed clinical integration using an approach outlined by Hall et al. [16] that highlights using the exam to identify the fives Es (effusion, ejection, equality, entrance, and exit) of cardiac ultrasound. 

Students were assigned to show up at a specific time and instruction occurred using the following method: students were given a quick response (QR) code, which led them to a 10-minute video that reviewed how to operate a Butterfly iQ ultrasound device (Butterfly Network, Inc. Guilford, Connecticut, United States) and perform a focused cardiac exam [17]. This instruction was part of a larger lab where students also learned how to perform a gallbladder, renal, and aorta exam. Immediately after watching the video on the cardiac ultrasound exam, students practiced acquiring normal images on a live simulated patient model one-on-one with an expert instructor. Due to time limitations, students acquired PSLA and subcostal four-chamber views. They were given 10 minutes to complete these two views and if time allowed, they practiced acquiring an apical four-chamber and parasternal short axis view as well. Instructors consisted of emergency medicine faculty with fellowship training in POCUS and diagnostic medical sonographers with formal echocardiography training. Instructors also discussed pathologic findings including where fluid would collect for a pericardial effusion, how to assess left ventricular ejection fraction, and how to assess for right ventricular dilation.

Immediately after completion of the didactic videos and individual hands-on scanning, students in groups of two to four completed a simulated case using a SonoSim Trainer (SonoSim, Inc., Santa Monica, California, United States) on a mannequin. Using the pre-built SonoSim Live Scan Critical Care Case 7 with the pathology and diagnosis blinded on the simulator screen, students were given the following case introduction: A 40-year-old female, with a history of metastatic carcinoma, presents with fever, weakness, and dyspnea. Vital signs: Temperature=39 degrees Celsius, blood pressure=70/p mmHg, heart rate=76 beats per minute, respiratory rate=28 breaths per minute, room air peripheral capillary oxygen saturation (SpO2)=93%. Students were then asked by the instructor to perform a PSLA cardiac POCUS view, identify pathology, and diagnose the patient’s etiology for hypotension. Students were not given choices for possible causes of hypotension and had to come up with the diagnosis on their own. The simulated exam consisted of an ultrasound video of a large pericardial effusion with right ventricular diastolic collapse. Prior to scanning, SonoSim Live scan stickers were placed on a mannequin in the 4th-5th intercostal space left parasternal, subxiphoid, and the left 5th intercostal space in the anterior axillary line.

Students were allowed to work as a group. They were assessed using a standardized data collection form on their ability to obtain a PSLA image using a five-point quality scale (1=unable to obtain image, 3=able to obtain image with technical flaws, 5=excellent image quality). We recorded whether assistance was needed with probe placement, probe maneuvering, and identifying pertinent anatomy. Students were also assessed on their ability to identify a pericardial effusion and diagnose cardiac tamponade.

After students performed the assessment and data were recorded, the instructors demonstrated how to acquire a PSLA view, and discussed the anatomic and pathologic findings present on the simulator images.

Statistical analysis

Analysis included median and standard deviations. Differences between pre- and post-assessment responses were analyzed using a Chi-square test. P <0.05 were considered significant. We performed all statistical analyses using VassarStats (©Richard Lowry, VassarStats: Website for Statistical Computation) [18] and Microsoft Excel (Microsoft Corporation, Redmond, Washington, United States).

## Results

We analyzed data on 132 pre-clerkship second-year medical students of which 126 (95%) had limited to no hands-on POCUS experience prior to the workshop (Table 1). Fifty-six (42%) students felt uncomfortable performing a four-view cardiac POCUS examination prior to instruction, 46 (35%) students were undecided on their comfort level, and 29 (22%) students were somewhat comfortable. Only one (0.7%) student felt very comfortable performing cardiac POCUS prior to instruction. Out of 132 students, 118 (89%) stated that they watched the pre-lab video that was a required part of their cardiovascular and hematology block course.

**Table 1:** Prior POCUS experience (pre-instruction), n=132 POCUS: point-of-care ultrasound

Experience	n (%)
I have not seen and have never used POCUS equipment	8 (6.1%)
I have only observed others performing POCUS, but have not practiced	24 (18.2%)
I have practiced POCUS (<5 times)	94 (71.2%)
I have practiced POCUS (5 or more times)	6 (4.5%)

A total of 108 (82%) students completed the post-workshop assessment and test. One hundred and five (97%) students indicated that their comfort level performing a four-view POCUS cardiac examination improved, with 55 students out of 108 (51%) stating that they felt significant improvement in their comfort level (Table 2). Three (3%) students indicated no change in comfort, and no students found that the instruction made them worse.

**Table 2:** Comfort level of students with performing a cardiac POCUS examination (pre-and post-instruction) POCUS: point-of-care ultrasound

Comfort level	n (%)
Pre-lab, n=132	
Very uncomfortable	21 (16%)
Somewhat uncomfortable	35 (26.5%)
Neither	46 (34.8%)
Somewhat comfortable	29 (22%)
Very comfortable	1 (0.8%)
Post-lab, n=108	
Significantly worsened	0 (0%)
Worsened	0 (0%)
No change	3 (2.8%)
Improved	50 (50.9%)
Significantly improved	55 (46.3%)

Comparing pre- to post-instruction knowledge (test) responses, we found significant improvement in the students’ ability to identify a pericardial effusion (46% to 69%) (p<0.001) on a PSLA cardiac image, and significant improvement in identifying the proper probe location and indicator direction for obtaining a subxiphoid view (54% to 85% (p<0.001). 

Of 57 student groups (132 students) who completed the post-workshop simulation case, 41 (72%) groups were able to adequately obtain a PSLA view on a simulator without needing guidance with probe placement or maneuvering from the instructor. Seven (12%) groups needed help with probe placement on the chest wall, and 12 (21%) groups needed help maneuvering the probe to obtain an adequate image. Three groups (5%) needed help with both probe placement and probe manipulation. 

Thirty-four student groups (60%) were able to identify the left ventricle, right ventricle, and pericardium on the simulator images; 35 groups (61%) made a correct diagnosis of pericardial effusion as the etiology for hypotension in the simulated patient, while 22 groups (39%) did not.

## Discussion

It is well established that students enjoy learning POCUS in UME and feel that POCUS should be integrated longitudinally into a UME curriculum [5,6,8,19]. However, little is known about the translation of POCUS learning into impact on patient care. Very few studies assess students’ ability to utilize POCUS within a clinical context, especially in those students who are in their pre-clinical years and novice to ultrasound [8]. This study was novel in that we evaluated the ability of pre-clinical students to incorporate cardiac ultrasound findings in the evaluation of a hypotensive patient using a simulator. These students were novices to POCUS and cardiac pathology, yet a majority of students (61%) were able to make the correct diagnosis in a hypotensive patient after limited instruction and hands-on scanning. In addition, we found that 72% of student groups (roughly 95 out of 132 students) were able to acquire high-quality PSLA images on a simulator without the help of an instructor. In addition, we found the majority of students, 60% of student groups (roughly 79 students), were able to identify pertinent anatomy on the PSLA view including the left ventricle, right ventricle, and pericardium. Interestingly, only 19 student groups (roughly 44 students) were able to both acquire POCUS images and accurately interpret them, arriving at the correct diagnosis during this simulated case without any assistance from the instructor.

We found significant improvement in comfort and knowledge when comparing pre-instruction to post-instruction, which is similar to many prior studies assessing POCUS in UME [7,8,14].

We found that 28% of students were unable to acquire a PSLA image on a simulator, and even more students (39%) were unable to correctly identify a pericardial effusion and diagnose cardiac tamponade, which was the etiology of the simulated patient’s hypotension. There are multiple explanations for why some students were unable to learn this skill; the first reason being the amount of time and instruction needed to learn the skills required to acquire images for a cardiac ultrasound examination may vary and cardiac POCUS is one of the harder examinations to perform [20]. Additionally, it is reasonable to conclude that 10 minutes of one-on-one hands-on instruction and 35 minutes of video is simply not enough time for some students to learn the complexities of acquiring and interpreting cardiac ultrasound. While there are limited proficiency studies assessing cardiac ultrasound [20], there are society guidelines through the American College of Emergency Physicians recommending at least 25-50 examinations be performed to attain proficiency in transthoracic cardiac ultrasound [21]. During the post-instruction simulated case, students were only asked to acquire a PSLA view. This single view as opposed to a full four-view cardiac ultrasound could have negatively impacted their ability to fully recognize pathology. It is less likely that the training was not effective as students showed improved knowledge and comfort with cardiac ultrasound. Lastly, pre-clinical students have not had exposure to pathology, including patients with hypotension and cardiac tamponade, outside of the classroom setting, which likely had an impact on their ability to recognize this diagnosis in a clinical scenario.

Lum et al. assessed the ability of 64 medical students, during their clinical years, to integrate lung ultrasound into a simulated case of patients with undifferentiated dyspnea [14]. Nine student groups were evaluated, of which correctly four identified pulmonary edema on lung ultrasound. Although this study differed from ours regarding sample size and methodology, we did find similar results in that knowledge of POCUS improved after a short didactic and hands-on scanning session. Similarly, we found that knowledge of POCUS, including how to acquire and interpret images, did not necessarily translate clinically for all students. This is because they failed to use it in a clinical scenario when POCUS is known to be helpful or they failed to identify pathology. While we know ultrasound is a useful tool to differentiate acute heart failure from chronic obstructive pulmonary disease (COPD) [22], in Lum et al.’s study they found that students were less likely to use ultrasound in patients with suspected COPD. Similarly, in our study, some students were unable to translate skills with POCUS into a correct diagnosis.

As we continue to integrate POCUS into UME nationally, it is important to better understand what type of training and experience is needed to translate POCUS education into a clinical scenario. More studies are needed, including simulated and within the clinical setting, to further assess the impact of POCUS education on diagnosis and management strategies. Studies such as this can serve to help guide curriculum design.

Limitations

Although the knowledge tests assessed students individually, students completed the simulated case in groups of two to four. This was due to curriculum time restrictions, resource limitations, and social distancing guidelines due to the COVID-19 pandemic. Because of these limitations, we were unable to assess the ability of individual students to acquire and interpret POCUS images and independently assess their ability to use cardiac POCUS within a simulated clinical scenario. Thus, they worked as a group to acquire images and make a diagnosis, similar to a prior study [14]. It is possible that some students in the groups that had correct imaging techniques and responses may not have been able to perform or interpret a POCUS cardiac examination independently. 

In addition, not all students participated in both the pre- and post-knowledge assessments. This introduces bias as it is possible the students who felt more confident or enjoyed the session may have been more likely to respond and could have inflated the number of correct responses. Not all students completed the required pre-lab video, and these 14 students were likely to be unfamiliar with recognizing cardiac pathology, including pericardial effusion. This could account for a lower number of student groups reaching the correct diagnosis if these students happened to be in the same group. However, we did not track this. 

Assessing psychomotor skills and the ability to make a diagnosis on a simulator can be different from doing the same on a patient in the clinical setting. Probe maneuvering on a simulator may not truly correlate with how one obtains a PSLA image in clinical practice. This in turn could impact students' identification of cardiac pathology. Future studies are needed to assess the ability of students to detect pathology in the clinical setting. Lastly, as these were pre-clinical medical students, we did not assess treatment and only evaluated whether they could make the correct diagnosis.

## Conclusions

In conclusion, after short, focused didactics and one-to-one hands-on instruction, most students were able to obtain high-quality PSLA images on a simulator without needing guidance from an instructor. When evaluating students’ ability to integrate POCUS into a simulated clinical scenario, we found that most students were able to identify a pericardial effusion and diagnose cardiac tamponade in a hypotensive patient; however, 39% were unable to determine the correct diagnosis.
